# Association between Child Abuse, Depression, and School Bullying among Chinese Secondary School Students

**DOI:** 10.3390/ijerph20010697

**Published:** 2022-12-30

**Authors:** Liu Liu, Xiaotao Wang, Binli Chen, Wing-Hong Chui, Xiying Wang

**Affiliations:** 1 School of Social and Behavioral Sciences, Nanjing University, Nanjing 210023, China; 2 School of Social Development, Nanjing Normal University, Nanjing 210097, China; 3 School of Social Development and Public Policy, Beijing Normal University, Beijing 100875, China; 4 Department of Applied Social Sciences, The Hong Kong Polytechnic University, Hong Kong, China; 5 Institute for Education Theories, Faculty of Education, Beijing Normal University, Beijing 100875, China

**Keywords:** school bullying, child abuse, depression, secondary school students, China

## Abstract

Introduction: School bullying is a serious social and public health problem. This article aims to explore the association between experiences of childhood abuse and involvement in school bullying, especially considering the mediating effects of depression. Methods: The sample of this study was 3059 students (1584 males and 1475 females) aged from 12 to 20, in eleven Chinese secondary schools, which included six middle schools, four high schools, and one vocational high school in both urban and rural areas. The multinomial logit regression and linear regression were conducted by the two gender groups, to determine the mediating effect of depression in the association between child abuse experiences and involvement in school bullying. Results: This study indicated that female students were less likely to be involved in school bullying. Male students were more represented in the bully-victim group and experienced more physical and mental abuse during childhood. Depression was a mediator between childhood abuse and involvement in school bullying. Nevertheless, there was a gender difference in that depression fully mediated the relationship between the two variables among female students, while it only partially meditated such a relationship for male students. Conclusions: These findings indicate that more school-based service interventions and evidence-based research are needed to more thoroughly investigate school bullying and its predictors in China.

## 1. Introduction

School bullying is a serious social and public health problem [1]. It is typically defined as a specific form of deliberate and aggressive behavior initiated by one or more students with a clear intention to cause discomfort or harm to another student or students [2]. This kind of behavior is characterized by intentionality, repetition, and a disparity of power between perpetrators and victims [3,4,5]. Bullying behavior can be covert (exclusion, spreading rumors) or overt (verbal and physical abuse) [6], which can include physical (hitting, kicking, pushing), verbal (teasing), relational (exclusion, isolation, humiliation in public), and/or cyber (hostile, aggressive messaging using mobile phone or the Internet) forms of aggression [2,7]. Four groups can be distinguished based on the different types of student involvement in bullying behaviors: (1) pure bullies, who bully others only; (2) pure victims, who are victimized by bullies; (3) bully-victims, who both bully others and are victims of others’ bullying; and (4) neutral students or bystanders, who are not involved in bullying activities [8,9,10,11].

Globally, almost one in three students (32%) has been bullied by their peers at school at least once in the last month [12], and it is estimated that 246 million children and adolescents experience school violence and bullying in some form every year [13]. School bullies, victims, and bully-victims, compared with the uninvolved neutral group, have been found to have more health and behavioral problems [14].

In Chinese society, school bullying was only recently recognized as a serious, pervasive societal issue [11], and China’s Ministry of Education issued several official documents from 2016 to 2018 that called for the prevention of school bullying throughout the nation. Chen and Zhi found, in their study, that 22.5% of Chinese secondary school students reported being bullied several times in the previous month, which was similar yet a little bit higher than the average prevalence rate of 18.5% in other OECD countries [15]. More male students than females were found to be both perpetrators and victims of bullying [15,16,17,18]. Most Chinese studies on school bullying have focused on the prevalence rate [19], risk and protective factors [20], negative consequences and serious implications [21], and potential interventions [19], but only a few studies have attempted to understand the relationship between school bullying and child abuse and explore its mediating factors.

One important interfering factor that can increase the students’ risk of becoming involved in school bullying is the experience of being abused during childhood [7,22]. According to a meta-analysis study’s results, “both victims and those who both bully and are victims (bully/victims) were more likely to be exposed to negative parenting behavior including abuse and neglect and maladaptive parenting” [23]. Researchers have estimated that in China, one in three Chinese people have experienced physical abuse, one in five experienced mental abuse, and two in five experienced neglect in their childhood [24,25]. The lifetime prevalence of child physical abuse in China was estimated at 36.6%, which was significantly higher than other countries in Asia and internationally [25,26]. Therefore, scholastic attention is very much needed on the linkage between childhood abuse and school bully involvement in Chinese society.

Hong et al. argued that depression is a potential mediator of the association between the experience of childhood abuse and involvement in school bullying [27]. Physically or mentally abused children report high levels of depression in childhood [28,29], and also in their adolescent and adult years [30,31,32]. Moreover, depression is a common mental health condition among school bullies and their victims [33,34,35], and it is a predictor of school bullying victimization [33,36]. In general, male students are more likely to engage in school bullying than female students [7]; researchers have found that male students were four to five times more likely to be bullies or bully-victims [37,38]. However, history shows that child abuse has been more prevalent among females [39]. According to a study by Lee et al. using a U.S. sample, compared to men, women had a greater likelihood of experiencing mental and sexual abuse, while there was no significant difference between genders with regard to physical abuse [40]. Moreover, female adolescents were generally found to have more depressive symptoms than males [41].

To fill the gap in the literature, this study aimed to explore the relationship between child abuse and school bullying among Chinese secondary school students, as well as to investigate the mediating effects of depression. To be more specific, based on the above mentioned empirical results, we hypothesize that being abused in childhood is related to a higher possibility of becoming both a bully and/or a bully-victim in school. Depression is hypothesized to mediate the relationship between these two variables: students who experience childhood abuse and depression are assumed to have an increased likelihood of involvement in school bullying. Notably, we try to explore school bullying and its connections with childhood abuse and depression among male and female students, respectively, at Chinese secondary schools, since males and females have distinct characteristics in both school bully and child abuse experiences.

## 2. Materials and Methods

### 2.1. Sample and Procedure

This study was part of an extensive quantitative survey we implemented in early 2019 exploring Chinese secondary school students’ lives and their experiences of school bullying. In the Chinese educational system, secondary school includes two levels, middle school and high school (including vocational high school), and each level has three grades. Middle school students usually range between 12 and 15 years old, and high school ones are generally aged from 15 to 18. The research protocol was reviewed and approved by the corresponding author’s university before data collection began. Administrators of the secondary schools, where the data were collected, also acknowledged the participants’ protection under the Human Subjects Protocol.

We identified participants of the survey through a stratification sampling approach in 11 Chinese secondary schools, which included 6 middle schools, 4 high schools, and 1 vocational high school in both urban and rural areas. Four to five classes in each of these secondary schools were randomly selected and all students in the selected classes were invited to participate. The students self-administered the questionnaires, which included items on school bullying, child abuse experiences, and depressive symptoms, within 30 to 45 min. Researchers were in the field to provide help to the participants if they wanted to seek clarification, especially the explanation on the academic terms. We briefly introduced the research purpose to the participants and emphasized their anonymity and confidentiality. They were allowed to decline participation by leaving the questionnaires blank with no adverse circumstances. We distributed a total of 4000 questionnaires and 3531 students completed them, with a response rate of 88.3%. The final sample of 3059 students, after list-wise deleting any responses that were missing data, consisted of 1584 male students (51.8%) and 1475 female students (48.2%). The average age of the participants was 14.68, and the median age was 15, ranging from 12 to 19.

### 2.2. Measures

We measured school bullying and victimization using the Chinese version of the Illinois Bully Scale (IBS) [20,42]. The participants were required to report their experience of bullying or victimization in the previous 30 days by a Likert-type scale ranging from 0 to 4 (never, one or two times, three or four times, five or six times, and seven or more times). Nine items, including “name-calling”, “rumor-spreading”, “teasing”, and “social exclusion”, formed the bullying subscale and assessed the students’ bullying behavior. Another four items, including “being teased”, “being called names”, and “being hit and pushed by others”, formed the victimization subscale and assessed the students’ victimization experiences. A higher score on both subscales indicated that the respondents had a greater likelihood of engaging in bullying behaviors or being bullied by others. The internal consistency of the bullying and victimization subscales was acceptable (bullying subscale: Cronbach’s α = 0.80; victimization subscale: Cronbach’s α = 0.77) in this study.

We measured child abuse experiences using two dummy variables, indicating whether the students, in their childhood, had ever been abused physically (Yes = 1; No = 0) or mentally (Yes = 1; No = 0).

To assess depression severity, we used the Chinese version of the Zung Self-Rating Depression Scale (SDS) [43], which is a short, widely used, comprehensive, self-administered instrument. The SDS features 20 items that are scored on a 4-point Likert-type scale ranging from 1 (rarely) to 4 (almost always). Thus, the scale scores range between 20 and 80, with higher scores indicating a more severe level of depression. Multiplying the score by 1.25 results in a total score out of 100. Four categories regarding respondents’ level of depression were clustered based on the total score: normal range (under 50), mild depression (between 50 and 59), moderate depression (between 60 and 69), and severe depression (70 and above) (Fried, 2017). This scale also had an acceptable internal consistency (Cronbach’s α = 0.73) in this study.

Moreover, we included several sociodemographic variables in the questionnaire relating to gender, BMI, household register, parents’ marital status, monthly allowance, and sexual orientation. We also included questions about whether the students had failed to pass exams, and whether they consumed cigarettes and alcohol.

### 2.3. Statistical Analysis

We first used the data to determine the grouping of the students into the four bullying categories—bullies, victims, bully-victims, and bystanders—as well as their level of depression and whether they had experienced child abuse. Because there were differences in gender in bullying and victimization [16,17], we distinguished different gender groups and tested the gender differences for all of the variables. Later, we conducted multinomial logit regression and linear regression by the two gender groups, to determine the mediating effect of depression in the association between child abuse experiences and involvement in school bullying. The KHB method was used to test the significance of the mediating effects [44]. Statistical analyses were conducted using STATA 16.0 software.

## 3. Results

### 3.1. Descriptive Analyses

In general, the prevalence rate of bullying perpetration in this study was 13.9%, the victimization rate was 18.7%, and 19.3% of students were both bullies and victims (see Table 1). In line with prior research [8,11], student participants were classified into four groups—bullies, victims, bully-victims, or bystanders—based on their self-reported experiences of bullying and victimization. The cutoff point of bullying and/or victimization experiences was “three or four times” [9]. Namely, if a student reported three to four times or more of at least one behavior indicative of bullying and/or victimization, they were classified as bullies, victims, or bully-victims; the remainder of the students were classified into the bystander group.

We found a significant gender difference, according to the four-group classification, in the students’ bullying/victimization experiences. Comparatively, female students were less likely to be involved in school bullying activities. Among those who were involved, male students were more represented in the bully-victim group, while female students had a greater likelihood to be victims. We were surprised that female students were comparatively more likely to be included in the pure bully group than male students, who presented more in the bully-victim group.

There were significant gender differences in students’ child abuse experiences and depression. Male students were more likely to be both physically and mentally abused during childhood than female students. However, female students had significantly higher scores in the SDS than males, although few students indicated severe depressive symptoms.

Among the covariates, there were no gender differences in BMI, household register, and parents’ marital status, while significant gender differences appeared in passing exams, monthly allowance, sexual orientation, and experiences of smoking and drinking. Male students had comparatively better academic performance, more monthly allowance, and more experiences of smoking and drinking but were less represented in sexual orientation as homosexual or bisexual.

### 3.2. Regression Analyses

Because male and female students have different experiences of school bullying and victimization, we wanted to determine the relative effects of childhood abuse on students’ bullying and victimization with depression as the mediator (see Table 2 and Table 3). We conducted a classic three-step regression approach [45]: (a) in Model 2-1 and 3-1, we chose to use childhood abuse experiences to predict the level of depression severity, which is the mediator; (b) in Model 2-2 and 3-2, we used childhood abuse experiences to predict involvement in school bullying/victimization, which is the dependent variable; and (c) in Model 2-3 and 3-3, we used the regression measures of both the depression severity level and childhood abuse experiences to predict involvement in school bullying/victimization.

The results for male students are presented in Table 2. In Model 2-1, after we controlled for all sociodemographic variables, we found that experiencing childhood abuse had significant effects on the severity of male students’ depression. Holding all other variables constant, experiencing physical and/or mental childhood abuse increased the likelihood of experiencing depression for male students.

In Model 2-2, childhood abuse experiences also had a statistically significant effect on school bullying/victimization. The male students who experienced physical childhood abuse were 2.34 times more likely to be victims and 1.95 times more likely to be bully-victims, compared with the bystanders. Meanwhile, those who suffered mental childhood abuse were 2.77 times more likely to be bullies, 2.05 times more likely to be victims, and 3.39 times more likely to be bully-victims compared with the bystanders. The results suggest that childhood abuse experiences (both physical and mental) can increase the risk of involvement in school bullying/victimization.

In Model 2-3, as expected, the severity of depression was significantly related to male students’ involvement in bullying/victimization. If the severity level of male students’ depression increased by 1 unit, they were 1.02 times more likely to be bullies, 1.03 times more likely to be victims, and 1.05 times more likely to be bully-victims, compared with the bystanders. Moreover, male students who experienced physical childhood abuse were significantly more likely to be victims (118% higher risk) and bully-victims (78.6% higher risk); while those who had been mentally abused during childhood were significantly more likely to be bullies (163% higher risk), victims (93.5% higher risk), and bully-victims (206% higher risk). The results suggest that childhood abuse experiences (both physical and mental) have direct and indirect (via depression severity level) effects on male students’ involvement in school bullying/victimization.

The KHB methods were used to examine the significance of the direct and indirect mediating effects. For male students, suffered from childhood physical and mental abuse would significantly directly and indirectly (via depression) affect the likelihood of being victims and bully-victims at secondary schools. More specifically, 7.71% of the effect of physical abuse and 9.89% of the effect of mental abuse on the risk of being victims, and 15.50% of the effect of physical abuse and 9.48% of the effect of mental abuse on the risk of being bully-victims were mediated by the depression severity level. However, male students’ childhood physical and mental abuse did not affect the risk of being bullies via the mediating effect of depression, while physical abuse did have a significant direct effect on the likelihood of becoming bullies.

We investigated the female students using the same three variable measures as reported in Table 3. Different from Model 2-1 and male students, experiencing physical childhood abuse was not significantly related to female students’ depression severity level in Model 3-1. However, female students who had experienced mental abuse presented 3.46 times higher levels of depression severity compared with those who had not been mentally abused.

In Model 3-2, female students who had experienced mental abuse were significantly more likely to be victims (68.2% higher risk) or bully-victims (97.4% higher risk). However, there was no significant relationship between female students who had experienced physical abuse and their involvement in school bullying/victimization.

In Model 3-3, after adding both the depression severity level and childhood abuse experiences in the model, neither physical nor mental childhood abuse experiences showed a statistically significant relation to female students’ involvement in school bullying/victimization. Nevertheless, the severity of depression was still significantly related to female students’ involvement in bullying/victimization. When the score for female students’ depression severity level increased by 1 unit, female students were 1.04 times more likely to be victims, and 1.08 times more likely to be bully-victims, compared with the bystanders.

The results of Table 3 suggest that female students’ childhood abuse experiences affected their involvement in school bullying/victimization via depression differently than male students. Physical abuse experiences had no significant effect on female students’ involvement in school bullying or victimization, and mental abuse experiences had only indirect effects on female students’ involvement in school bullying/victimization, with depression as the mediator.

Similarly, we used the KHB method to test the mediating effects of depression that are suggested in Table 3. The results showed that, for female students, the mediating effect of depression between physical abuse and the involvement in school bullying/victimization was not significant. In another word, physical abuse experiences had neither direct nor indirect (via depression) effects on female students’ school bullying and victimization. However, 24.9% of the effect between mental abuse and the likelihood of becoming victims at schools, and 42.06% of the effect between mental abuse and the likelihood of being bully-victims, were significantly mediated by depression among female students. At the same time, the direct effects of mental abuse were not significant.

## 4. Discussion

Using a sample of 3059 Chinese secondary school students in this study, we provided prevalence rates of school bullying perpetration and victimization, and some measures of influence from mediating factors. It also revealed gender differences in the relationships between involvement in school bullying/victimization, childhood abuse experiences, and the severity of depression. In accordance with previous research [19,27], we found that male students were more likely to be involved in school bullying/victimization. However, the findings further revealed that if students were divided into four groups—bullies, victims, bully-victims, and bystanders—female students had a greater likelihood to be pure bullies than male students, while male students were comparatively more likely to be included in the bully-victim group. Therefore, male students tend to be more involved in school bullying activities, both as bullies and victims of bullies. In addition, inconsistent with a prior U.S. study [40], male students in this current study had experienced more physical and mental abuse, which might reflect cultural differences: Chinese parents tend to be stricter with their sons than their daughters [46].

In accordance with prior research findings and the hypothesis of this study, childhood abuse is closely related to involvement in school bullying, especially with being victims. Criminologists argue that victimization is a process combining a set of factors, including both environmental and individual ones, rather than a random process [47,48]. Abuse experience is thus confirmed in this study as one of the individual characteristics that may lead to school bully victimization. A growing body of literature has demonstrated that children who are physically or mentally abused at home or out of school are more likely to be submissive rather than defend themselves in a violent situation to maintain their safety, which can render them more vulnerable to bullies and bullying in school [23,49]. Beyond bullying victimization, bullying perpetration is another outcome of child abuse [50,51,52], which is also confirmed through the results of this study, especially for the male students. Abused children have a higher likelihood of exhibiting aggressive behaviors and are more inclined toward bullying behaviors [23,27]. This finding is also in accordance with the theoretical concern of the “victim to offender cycle” that suggests the experiences of childhood maltreatment would be a potential influencing factor that drives individuals to be involved in later delinquent or even criminal activities [53].

In addition, we found that depression was a mediator between the experience of child abuse and involvement in school bullying [27]. Furthermore, this study indicated that there was a gender difference, which made the findings of this study unique compared to the existing ones. The results of this study showed that physical and mental abuse could have direct and indirect (via depression) effects on boys, while, more subtly for girls, physical abuse did not have an impact and mental abuse had only an indirect effect. This difference might be caused by the Chinese cultural background, as Chinese families have been characterized as patriarchal [54]. Therefore, further research should investigate the family structure and pay more attention to the young people’s social networks.

Clinical implications reflecting the findings of this study are thus recommended. Social work interventions and school counseling services are generally recommended for improving Chinese secondary school students’ mental health, especially those who have experienced physical and/or mental abuse, to reduce the possibility that they become involved in school bullying. Information about the specific experiences of childhood abuse may help teachers and service providers identify individuals who are at risk of developing depression [55]. Therefore, a routine inquiry concerning students’ childhood abuse could alert social workers to provide help to these students, thereby decreasing the severity of any depression they might suffer and hopefully dissuade them from involvement in school bullying. In addition, the interventions and services focusing on students’ mental health may be more productive with female students, as this study showed that depression fully mediated child abuse experiences and school bullying for female students.

This study is not without limitations. The first limitation is concerned with the measurement of childhood abuse experiences. The “yes” or “no” response cannot assess the severity of the abuse that students experienced. In addition, this study relied on self-reported data, which may lead to response bias and social desirability bias [56]. Thus, we may have underestimated the prevalence of childhood abuse experiences, depression severity, and involvement in school bullying. Furthermore, although the current study has valuable findings on the relationships between childhood abuse experiences, depression severity level, and school bullying involvement, we were unable to reveal the causal relationships between the above variables, as it was a cross-sectional study rather than a longitudinal one.

## 5. Conclusions

In conclusion, with a large and representative sample of secondary school students, this study achieved the research goal by providing reliable estimates of the prevalence of school bullying in China and demonstrated that depression mediated the relationship between childhood abuse and involvement in school bullying. The present study also confirmed the significant differences between genders regarding both involvement in school bullying and depression’s mediating effects on the two variables. These findings not only contribute to the existing literature, but also indicate that more school-based service interventions and evidence-based research are needed to more thoroughly investigate school bullying and its predictors.

## Figures and Tables

**Table 1:** Descriptive Statistics for Males (n = 1584) and Females (n = 1475).

	Total	Male	Female	*p*
Bully status				***
Bully (%)	13.90	12.75	15.05	
Victim (%)	18.70	19.19	18.37	
Bully-Victim (%)	19.30	22.79	15.32	
Bystanders (%)	48.10	45.27	51.25	
Abuse: Physical abuse				***
Yes (%)	9.0	12.18	5.69	
No (%)	91.0	87.82	94.31	
Abuse: Mental abuse				**
Yes (%)	14.0	16.04	11.80	
No (%)	86.0	83.96	88.20	
Depression (M)	41.91	41.28	42.60	***
Normal range (%)	38.60	41.00	36.00	
Mild depression (%)	37.40	36.30	38.50	
Moderate depression (%)	20.80	20.50	21.20	
Severe depression (%)	3.20	2.20	4.30	
BMI (M)	20.03	20.15	19.90	n.s.
Household register				n.s.
Rural (%)	39.20	39.52	39.19	
Urban (%)	60.80	60.48	60.81	
Passed exams or not				**
Yes (%)	31.20	33.71	28.68	
No (%)	68.80	66.29	71.32	
Parents’ marriage status				n.s.
Married (%)	88.90	88.83	88.88	
Other (%)	11.10	11.17	11.12	
Monthly allowance (RMB, M)	451.33	505.14	390.02	*
Sexual orientation				***
Heterosexual (%)	64.60	66.54	62.37	
Homosexual/Bisexual (%)	8.00	5.62	10.51	
Exploring (%)	27.40	27.84	27.12	
Smoking				***
Yes (%)	7.40	10.04	4.47	
No (%)	92.60	89.96	95.53	
Drinking				***
Yes (%)	41.80	47.10	36.07	
No (%)	52.20	52.90	63.93	

Note. *, *p* < 0.05; **, *p* < 0.01; ***, *p* < 0.001; n.s., *p* > 0.05; *p* refers to the tests for gender differences.

**Table 2:** Logit and OLS Regression Predicting Category of Bully Status and Depression for Males (n = 1584).

	Model 2-1		Model 2-2						Model 2-3					
	Depression		Bully		Victim		Bully-Victim		Bully		Victim		Bully-Victim	
	Coefficient	*SE*	Coefficient	*SE*	Coefficient	*SE*	Coefficient	*SE*	Coefficient	*SE*	Coefficient	*SE*	Coefficient	*SE*
Constant	40.60 ***	1.36	−0.96	0.56	−1.05	0.55	−0.48	0.49	−2.01 **	0.76	−2.31 **	0.71	−2.52 ***	0.66
Abuse														
Physical abuse (No = 0)	2.28 ***	0.61	0.15	0.31	0.85 **	0.25	0.67 **	0.24	0.09	0.31	0.78 **	0.25	0.58 *	0.24
Mental abuse (No = 0)	2.42 ***	0.55	1.02 ***	0.25	0.72 **	0.23	1.22 ***	0.21	0.97 ***	0.25	0.66 **	0.23	1.12 ***	0.21
Depression									0.02 *	0.01	0.03 **	0.01	0.05 ***	0.01
BMI	0.03	0.04	0.00	0.02	−0.02	0.02	−0.01	0.02	0.00	0.02	−0.02	0.02	−0.01	0.02
Household register (Rural = 0)	−0.85 *	0.43	−0.32	0.20	−0.22	0.17	−0.14	0.17	−0.29	0.20	−0.19	0.17	−0.10	0.17
Passed exams or not (No = 0)	−1.55 ***	0.41	0.06	0.19	−0.01	0.16	−0.32 *	0.16	0.09	0.19	0.03	0.16	−0.25	0.17
Parents’ marriage status (Other = 0)	−1.22 *	0.56	0.23	0.28	0.07	0.23	−0.04	0.21	0.26	0.28	0.11	0.23	0.02	0.21
Monthly allowance (yuan)	0.00	0.00	0.00 *	0.00	−0.00	0.00	0.00 *	0.00	0.00 *	0.00	−0.00	0.00	0.00	0.00
Sexual orientation (Heterosexual = 0)														
Homosexual/Bisexual	2.14 **	0.79	0.58	0.36	0.61	0.33	0.66 *	0.30	0.55	0.36	0.56	0.33	0.58	0.30
Exploring	1.30 **	0.42	0.14	0.20	−0.16	0.17	−0.10	0.16	0.11	0.20	−0.20	0.17	−0.16	0.17
Smoking (Yes = 0)	0.21	0.64	−0.72 *	0.28	−0.05	0.28	−0.56 *	0.24	−0.73 **	0.28	−0.07	0.28	−0.57 *	0.24
Drinking (Yes = 0)	−0.50	0.39	−0.20	0.18	−0.20	0.16	−0.34 *	0.15	−0.19	0.18	−0.19	0.16	−0.33 *	0.15
LL/R-Squared	0.122				−1892.62				−1880.22					

Note. *, *p* < 0.05; **, *p* < 0.01; ***, *p* < 0.001. Dummy variables representing the schools are also included in the models.

**Table 3:** Logit and OLS Regression Predicting Category of Bully Status and Depression for Females (n = 1475).

	Model 3-1		Model 3-2						Model 3-3					
	Depression		Bully		Victim		Bully-Victim		Bully		Victim		Bully-Victim	
	Coefficient	*SE*	Coefficient	*SE*	Coefficient	*SE*	Coefficient	*SE*	Coefficient	*SE*	Coefficient	*SE*	Coefficient	*SE*
Constant	45.65 ***	1.58	0.92	0.67	−0.00	0.63	−0.07	0.63	0.13	0.84	−1.76 *	0.79	−4.01 ***	0.86
Abuse														
Physical abuse (No = 0)	0.77	0.91	0.53	0.38	0.56	0.35	0.58	0.36	0.54	0.38	0.55	0.35	0.57	0.37
Mental abuse (No = 0)	3.46 ***	0.66	0.10	0.28	0.52 *	0.25	0.68 **	0.26	0.05	0.29	0.39	0.26	0.39	0.27
Depression									0.02	0.01	0.04 ***	0.01	0.08 ***	0.01
BMI	0.00	0.04	−0.02	0.02	−0.01	0.02	−0.01	0.02	−0.02	0.02	−0.01	0.02	−0.01	0.02
Household register (Rural = 0)	−0.18	0.47	0.07	0.20	0.05	0.18	0.10	0.19	0.07	0.20	0.05	0.18	0.13	0.20
Passed exams or not (No = 0)	−2.67 ***	0.47	0.02	0.19	−0.25	0.18	−0.94 ***	0.22	0.06	0.19	−0.16	0.18	−0.74 **	0.23
Parents’ marriage status (Other = 0)	−0.97	0.60	−0.61 **	0.23	−0.45 *	0.23	−0.37	0.26	−0.61 **	0.23	−0.43	0.23	−0.28	0.26
Monthly allowance (yuan)	−0.00 *	0.00	0.00	0.00	−0.00	0.00	0.00	0.00	0.00	0.00	−0.00	0.00	0.00	0.00
Sexual orientation (Heterosexual = 0)														
Homosexual/Bisexual	1.70 **	0.64	−0.17	0.27	0.08	0.25	−0.13	0.29	−0.18	0.27	0.02	0.26	−0.32	0.30
Exploring	1.43 **	0.45	0.17	0.18	0.15	0.17	0.17	0.19	0.15	0.18	0.10	0.17	0.06	0.19
Smoking (Yes = 0)	−1.95 *	0.96	−1.37 **	0.40	−1.14 **	0.41	−1.51 ***	0.40	−1.35 **	0.40	−1.09 **	0.41	−1.42 ***	0.40
Drinking (Yes = 0)	−1.13 **	0.43	−0.26	0.18	−0.15	0.17	−0.40 *	0.18	−0.25	0.18	−0.12	0.17	−0.31	0.18
LL/R-Squared	0.120				−1702.55				−1673.34					

Note. *, *p* < 0.05; **, *p* < 0.01; ***, *p* < 0.001. Dummy variables representing the schools are also included in the models.

## Data Availability

Ethics approvals do not permit these potentially re-identifiable data to be made publicly available.
